# High-level Fusion Coupled with Mahalanobis Distance Weighted (MDW) Method for Multivariate Calibration

**DOI:** 10.1038/s41598-020-62396-y

**Published:** 2020-03-25

**Authors:** Qianqian Li, Zhisheng Wu, Ling Lin, Jingqi Zeng, Jixiong Zhang, Hong Yan, Shungeng Min

**Affiliations:** 1 School of Chinese Material Medica, Beijing University of Chinese Medicine, Beijing, 100029 China; 2 College of Science, China Agricultural University, Beijing, 100193 China

**Keywords:** Chemistry, Analytical chemistry, Cheminformatics, Theoretical chemistry

## Abstract

Near infrared spectra (NIR) technology is a widespread detection method with high signal to noise ratio (SNR) while has poor modeling interpretation due to the overlapped features. Alternatively, mid-infrared spectra (MIR) technology demonstrates more chemical features and gives a better explanation of the model. Yet, it has the defects of low SNR. With the purpose of developing a model with plenty of characteristics as well as with higher SNR, NIR and MIR technologies are combined to perform high-level fusion strategy for quantitative analysis. A novel chemometrical method named as Mahalanobis distance weighted (MDW) is proposed to integrate NIR and MIR techniques comprehensively. Mahalanobis distance (MD) based on the principle of spectral similarity is obtained to calculate the weight of each sample. Specifically, the weight is assigned to the inverse ratio of the corresponding MD. Besides, the proposed MDW method is applied to NIR and MIR spectra of active ingredients in deltamethrin and emamectin benzoate formulations for quantitative analysis. As a consequence, the overall results show that the MDW method is promising with noticeable improvement of predictive performance than individual methods when executing high-level fusion for quantitative analysis.

## Introduction

Gas chromatography^1^, high-performance liquid chromatography^2^, thin-layer chromatography^3^, gas chromatography-mass spectrometry, liquid chromatography-mass spectrometry^4,5^ are frequently-used techniques to detect the concentration of active ingredients in pesticides. While they may come across some inconveniences such as time-consuming, expensive lab-cost and complex pre-treatment. Admittedly, vibrational spectroscopic techniques respond fast and require no pre-treatment which are especially suitable for real-time measurement and rapid analysis^6,7^. NIR and MIR techniques have been widely used to determine the active ingredients and illegal added component in pesticide formulations^8–12^. The two commonly used techniques of NIR and MIR are generated by the transition of vibrational energy levels. The transition occurs only when it absorbs a certain wavelength. To be concrete, as for MIR, it mainly absorb the fundamental vibrations of C-H, N-H and O-H, whereas, NIR contains a wealthy information of the combinations and overtones of the fundamental vibrations of C-H, N-H and O-H.

Data fusion is a tool to combine the data originated from different sources comprehensively. To a certain degree, a single technique can only perceive limited partial information, therefore multiple techniques are expected to provide sufficient information from different viewpoints. Data fusion techniques^13–15^ have been extensively employed with the purpose of getting a more well-rounded result. Generally, data fusion strategies can be basically classified into three levels, i.e., low-level, mid-level and high-level data fusion^16^. When executing high-level fusion, individual models for each data source are developed first, and afterwards the final results are obtained by combining each individual model. The advantage of high-level fusion is that each matrix is treated independently and the result from inefficient technique is assigned to lower weight which will not affect the overall performance^17,18^. Accordingly, the technique with high predict performance is assigned to a large weight for fusion. In consequence, high-level fusion is mainly focusing on the particularities of each individual technique with the challenge of obtaining a better performed model. Multiple approaches have been applied to high-level data fusion such as majority vote^19^, Bayesian networks (BNs)^20^, and Dempstere-Shafer’s^21^ method. However, these methods are mainly focused on classification issues^22–27^ and have been less explored to quantitative analysis. In this study, we propose the Mahalanobis distance weighted (MDW) method to employ high-level fusion to quantitative analysis.

Mahalanobis distance (MD) was first proposed by P. C. Mahalanobis in 1938^28^. Employing MD on mathematical algorithm for chemical identity classification was described by Mark and Tunnell^29,30^. Based on the principle of spectral similarity, MD manifests its distance from the initial calibration set. Theoretically speaking, MD is considered as a measure of standard deviation, and the samples are expected to lie within three times the MD of their respective group means^29^. Therefore, a sample with large MD value might decrease the robustness and predictability of the model. Similarly, incorporating a sample with small MD value for modeling might increase the predictive ability. To be specific, a sample with large MD value indicates that it is farther from the calibration set in multidimensional space, and it is identified as the dissimilar one with the initial calibration set, and correspondingly it will give a relative poor prediction result when modeling. Comparably, a sample with a small MD value manifests that it is close to the calibration set in multidimensional space, and therefore it could be concluded that this sample is similar to the calibration set and will give a promising predictability.

The MDW method is proposed according to the MD value. It is worth noting that three times of the group means of MD is treated as a criterion. Moreover, if a sample (for a specific method) lies within the limits of criterion, the weight is given corresponding to the reciprocal of the MD value; otherwise, when the MD is over criterion value, the weight is assigned to zero. Since one sample owns a particular MD value from one technique, several MD values are acquired for one sample when executing fusion strategy. A sample is assigned to several weights by means of different methods. What is worth mentioning, the total weight is set as 1.

Integrating the individual results to get a final output by assigning each sensor a rational weight is the principle of high-level fusion. In views of the above, a weighted method upon MD is put forward to fuse each technique by taking all the individual results into account. In this study, the MDW method is applied to individual NIR and MIR spectroscopic analysis for active ingredient determination in deltamethrin and emamectin benzoate formulations. The performance of the three methods (individual NIR, individual MIR, MDW fusion) from deltamethrin and emamectin benzoate formulations are evaluated by the predict ability of the models.

## Theory and algorithms

### High-level fusion

In high-level fusion, the NIR and MIR matrices are performed to develop two individual models according to five-fold cross-validation of partial least square (PLS), respectively. In addition, the fusion results are calculated by combing the outputs of the two individual results. The main advantage of high-level fusion is to obtain a comprehensive utilization of all individual methods rather than take advantage of only one method. As a result, more accurate and credible results are gained with reasonable weights assigned to different samples on the basis of different methods.

In the high-level fusion, separate models are calculated by the corresponding data source, and the results are combined to acquire the final declaration^16^. In the fusion approach, the final result is obtained by associating the results of each method via their weights. The outputs of each sample can be represented by Eq. 1

1 $${y}_{p(x)}=\mathop{\sum }\limits_{i=1}^{L}{y}_{i(x)}{w}_{i(x)}$$

where *y*_*p*_ is the fusion result, *L* is the number of sensors, *w*_*i*_ and *y*_*i*_ are the weight and the predicted result for the *i*^th^ sensor, respectively. The output *y*_*p*_ is acquired by integrating all the individual results with their weights.

### Mahalanobis distance (MD)

The MD value is calculated on the basis of the distribution of the original samples^29^. The largest MD value sample is the most different one from the initial set. The MD of an observation *x* from a set of observations ***X***_***i***_ (the calibration set corresponding to sensor *i*) is defined as Eq. 2 in squared units.

2 $${D}^{2}(x)=(x-{\bar{X}}_{i}){\prime} {M}_{i})(x-{\bar{X}}_{i})$$

where ${\bar{X}}_{i}$ and ***M***_***i***_ are the mean and covariance matrix of ***X***_***i***_, respectively.

In order to reduce the dimensionality and eliminate the overlapping information in coexistence, the score matrix (***S***) is used to calculate MD to avoid collinear problems in ***M*** matrix. It is worth noting that, the corresponding score matrix ***S***_***i***_ (related to the calibration set of ***X***_***i***_ not for all the samples) is utilized to compute the MD values. Prior to obtain MD, the optimal dimension of PLS scores matrix is performed to acquire ***S***_***i***_.

### Mahalanobis distance weighted (MDW)

The weights are assigned to each sample according to MDW method. For each individual method, the score matrices are employed to calculate the MD. It is noteworthy that a sample is assigned to several weights by means of different detection methods.

**Figure 1:**
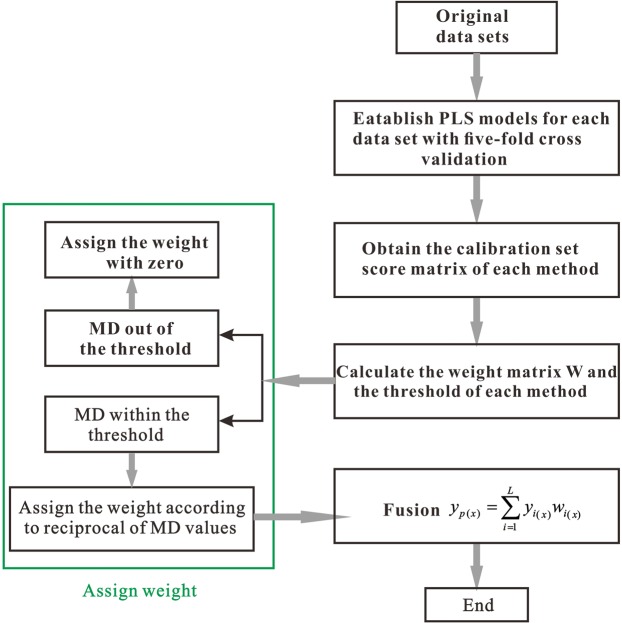
Scheme for explanation MDW approach for quantitative analysis.

The flowchart of high-level fusion combined with MDW method is illustrated in Fig. 1. In the first place, fit the PLS models for each data set and obtain the score matrices. The next step, calculate the distance for the samples in calibration set. And finally, compute the weight and the threshold. As shown in the schematization framework, each sample is assembled by different weights via MDW method. It can be summarized in the following steps:

1. A data matrix contains *n* samples in rows and *p* variables in columns.
2. Carry out PLS, the data are mean-centered before establishing regression model.
3. Obtain the root mean square error of cross-validation (RMSECV) and get the score matrices (***S***_***i***_) of the calibration set with the optimal latent variables (LVs) of each method.
4. The MD values are calculated and the weight matrix **W** (*n*_*v*_ × *L*) contains *n*_*v*_ samples (samples for validation) in rows and *L* (number of sensors) variables in columns.
5. Execute the individual results for high-level fusion by MDW method.
6. Get the final results generated by fusion method.

In the following sections, the characteristics and behaviors of the MDW method are discussed in detail.

### Model evaluation

Results from two individual methods are optimized at the stage of calibration set and then evaluated by RMSEP. In calibration set, the optimal LVs is determined by five-fold cross-validation method. As a matter of fact, an optimal model is formed with low root mean square error of prediction (RMSEP) and low bias. For the two parameters of RMSEP and bias, a better model is obtained following the principle: small RMSEP with small bias> small RMSEP with large bias > large RMSEP with small bias > large RMSEP with large bias.

### Software

The algorithms involved in this study are programmed by Matlab (Version 2016a, the MathWorks, Inc.). The coding scripts used in this study are available upon request.

## Data description

### Deltamethrin samples

Seventy-eight deltamethrin samples were prepared by technical deltamethrin (98.1%, obtained from Jiangsu Huangma Agrochemicals, China), dimethylbenzene (99.0%, Beijing Chemical Works, China) and commercial deltamethrin formulation emulsion (25 g/L, Bayer Crop Science, China). The exact concentration of deltamethrin in the commercial formulation was determined by high performance liquid chromatography (HPLC). The concentration of the samples were ranged from 0.1% to 4.98% (w/w). Details of the samples were shown in Table 1.

**Table 1:** The calibration and validation sets for deltamethrin and emamectin benzoate samples. Mean represents the mean value of the calibration or validation set, SD represents the standard deviation.

Data sets	All samples		Calibration set		Validation set	
	Mean (%)	SD (%)	Mean (%)	SD (%)	Mean (%)	SD (%)
Deltamethrin	2.5519	1.4418	2.5370	1.4446	2.6114	1.4761
Emamectin benzoate	1.4340	0.8188	1.4616	0.8244	1.352	0.8239

### Emamectin benzoate samples

Sixty emamectin benzoate samples were prepared by technical deltamethrin (73.1%, obtained from Jiangsu Huangma Agrochemicals, China), dimethylbenzene (99.0%, Beijing Chemical Works, China) and commercial deltamethrin formulation (1%, Beijing Yagoon Biological Pharmaceuticals, China). The exact concentration of emamectin benzoate in the commercial formulation was determined by HPLC. The concentration of the samples were ranged from 0.06% to 3.01% (w/w). Details of the samples were shown in Table 1.

### Near infrared (NIR) spectroscopy

The NIR spectra were acquired by the FT-NIR spectrometer (Spectrum One NTS, Perkin Elmer, USA) from 4000 cm^−1^ to 12500 cm^−1^ at a resolution of 4 cm^−1^, and the spectra were the mean value of 64 accumulations.

### Mid-infrared (MIR) spectroscopy

The MIR spectra were collected by the FT-IR spectrometer (Cary 630, Agilent, USA) with ATR accessory. The spectral range was between 650 cm^−1^ and 4000 cm^−1^ at a resolution of 4 cm^−1^ with 64 accumulations co-added.

## Result and discussion

### Influence of mahalanobis distance (MD) for high-level fusion

The physical meaning of MD is the normalized Euclidean distance (ED) in principal components space, and MD denotes the distance between a sample and the calibration set in multidimensional space. Three times of the training distribution centroid was treated as the threshold. As acknowledged, a sample with small MD value was thought as being consistent with the training distribution, and would give an acceptable result in return. Similarly, it was firmly convinced that a sample with large MD value would result in a worse predictive result theoretically. To investigate the weight of each sample, the following two cases were taken into consideration: within or out of criterion. On the one hand, if the MD value was out of the threshold, the weight was assigned to **0%**, since an abnormal sample would reduce the overall predictability dramatically. On the other hand, if the corresponding MD value was in the limits scope, the weight was given from **100%** to **0%** according to the reciprocal of MD. Specifically, to some extent, the MD value was negatively correlated with its weight, that is, it was related to the reciprocal of the weight when the MD value was confined in the criteria range.

### Spectra of deltamethrin and emamectin benzoate formulations

**Figure 2:**
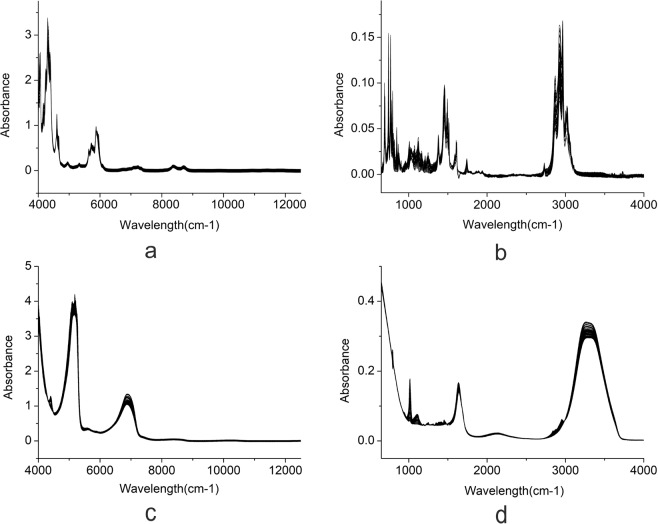
NIR and MIR spectra of deltamethrin and emamectin benzoate formulations. (**a**) NIR spectra of deltamethrin; (**b**) MIR spectra of deltamethrin; (**c**) NIR spectra of emamectin benzoate; (**d**) MIR spectra of emamectin benzoate.

The NIR and MIR spectra of deltamethrin and emamectin benzoate formulations were presented in Fig. 2. According to the NIR spectra of deltamethrin formulation (Fig. 2a), the spectral features of deltamethrin 4300 cm^−1^ and 4600 cm^−1^ were the combination of the stretching and bending vibrations of **C-H** and **N-H**, respectively. Moreover, 5900 cm^−1^ was ascribed to the first overtone of the stretching vibrations of **C-H**. In the MIR spectra of deltamethrin (Fig. 2b), the characteristic peaks of 700 cm^−1^ was associated with the bending vibration of **C-H** in the three-membered ring. The peaks at 750 cm^−1^ was corresponding to the deformation vibrations of **C-H** in benzene ring. As is acknowledged, the peak at 1123 cm^−1^ was ascribed to the stretching vibration of **C-O**. What is more, the characteristic peak of 1500 cm^−1^ was associated with the stretching vibration of **C-H** in the three-membered ring. In addition, the peak located at 1610 cm^−1^ in the region of 1600–1650 cm^−1^ was assigned to the stretching vibration of **C**=**C**. As far as is known, the peaks at 1720 cm^−1^ and 1740 cm^−1^ were ascribed to the bending vibration of **C**=**O**. The peaks at 3000 cm^−1^ was corresponding to the stretching vibrations of **C-H** in benzene ring.

On Fig. 2c (the NIR spectra of emamectin benzoate), the spectra located around 4400 cm^−1^ and 5200 cm^−1^ were the combination of the stretching and bending vibrations of **C-H** and **O-H**, respectively. And 6800 cm^−1^ was ascribed to the first overtone of the stretching vibration of **N-H**. In the MIR spectra of emamectin benzoate (Fig. 2d), the peak around 1020 cm^−1^ was ascribed to the stretching vibration of **C-O**. Moreover, the peak at 1640 cm^−1^ was ascribed to the bending vibration of **C**=**C**. Besides, the wide peak near 3300 cm^−1^ was associated with the stretching vibration of O-H.

### Deltamethrin formulation data

Monte-Carlo (MC) outlier approach was carried out upon running 1000 times for outlier detection. It turned out that no sample was kicked out after MC outlier detection approach. Afterwards, the samples were separated into calibration set (63 samples) and validation set (15 samples) according to the concentration from high to low. In this study, the spectra were corrected by autoscaling pre-treatment method to make sure each column had a mean of 0 and a variance of 1. Autoscaling was a mathematical transformation method to calculate the ratio of the mean centering spectra and the standard deviation spectra. As one of the data standard processing in factor analysis, it gave all variables a chance to be treated fairly regardless of the absolute concentration. As a result, all the wavelength variables were assigned to the same weight after autoscaling preprocessing.

**Figure 3:**
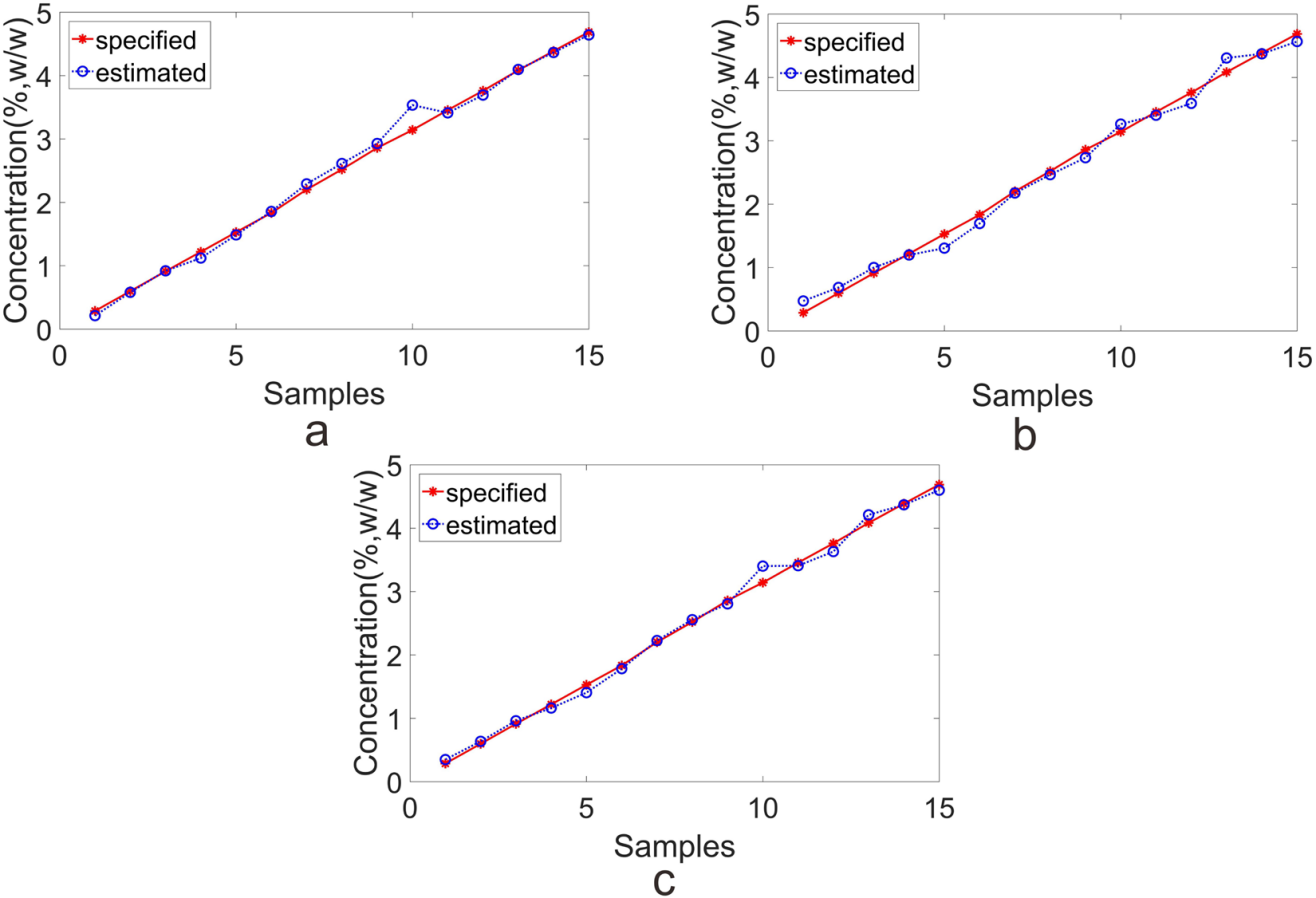
The plot of predicted and actual value in test set of deltamethrin samples. (**a**) individual NIR; (**b**) individual MIR; (**c**) MDW for fusion.

Five-fold cross validation technique was used to explore the predictive performance of each approach. It is generally known that the number of LVs was a critical parameter in calibration stage. As a result, ten and seven LVs were respectively chosen for NIR and MIR data set when executing PLS algorithm. After carrying out PLS algorithm, the plot of estimated and specified values in validation set were shown in Fig. 3a,b. The predicted results for each technique were summarized in Table 2. Since RMSEP and the bias were acknowledged as the model evaluation indicator, a better predictive ability was accompanied with small RMSEP and small bias. On the whole, the NIR method performed superior results than MIR method with smaller RMSEP and smaller bias.

**Table 2:** Results of MDW and individual methods on deltamethrin and emamectin benzoate data sets.

Pesticides	Methods	RMSEP (%)	Slop	Bias (%)
Deltamethrin	NIR	0.1164	1.0123	−0.0120
	MIR	0.1294	0.9772	0.0403
	MDW fusion	**0.1016**	0.9925	0.0195
Emamectin benzoate	NIR	0.1047	0.9527	0.0601
	MIR	0.0765	0.9886	0.0114
	MDW fusion	**0.0596**	0.9786	0.0205

The deltamethrin data matrix was implemented to investigate the effectiveness of MDW method for high-level fusion in the following steps. In the first place, the NIR and MIR approaches were performed PLS algorithm. Then the score matrix from each method was employed to calculate the MD and the threshold. Finally, the weight was obtained via the principals confined in Section 4.1. Based on the above procedure, MDW method was carried out to employ high-level fusion for quantitative analysis. The thresholds for the two methods were displayed in Table 3, which was the standard for assigning weight. As displayed in Table 3, there were no sample beyond their own threshold. Since all the samples were in the scope of threshold, the weights were assigned to the inverse relationship of the corresponding MD values.

**Table 3:** The MD of the validation set of deltamethrin and emamectin benzoate data sets from NIR and MIR methods. Threshold represents three times of the group mean value.

Sample NO.	Deltamethrin		Emamectin benzoate	
	NIR	MIR	NIR	MIR
1	2.731	2.454	5.463	4.092
2	2.157	1.904	3.267	2.244
3	2.213	2.112	7.631	1.206
4	3.441	3.017	3.468	1.633
5	2.886	3.489	8.283	1.692
6	2.183	2.661	2.229	2.155
7	3.758	2.961	3.055	1.953
8	1.523	2.310	1.899	1.892
9	2.92	1.700	4.962	1.723
10	3.7	3.808	1.914	1.829
11	2.624	2.925	3.183	0.883
12	1.9	1.254	2.947	1.531
13	2.135	1.843	3.459	1.785
14	2.477	1.797	3.086	1.411
15	2.423	1.870	2.613	1.922
Threshold	**8.239**	**7.517**	**9.154**	**5.705**

**Figure 4:**
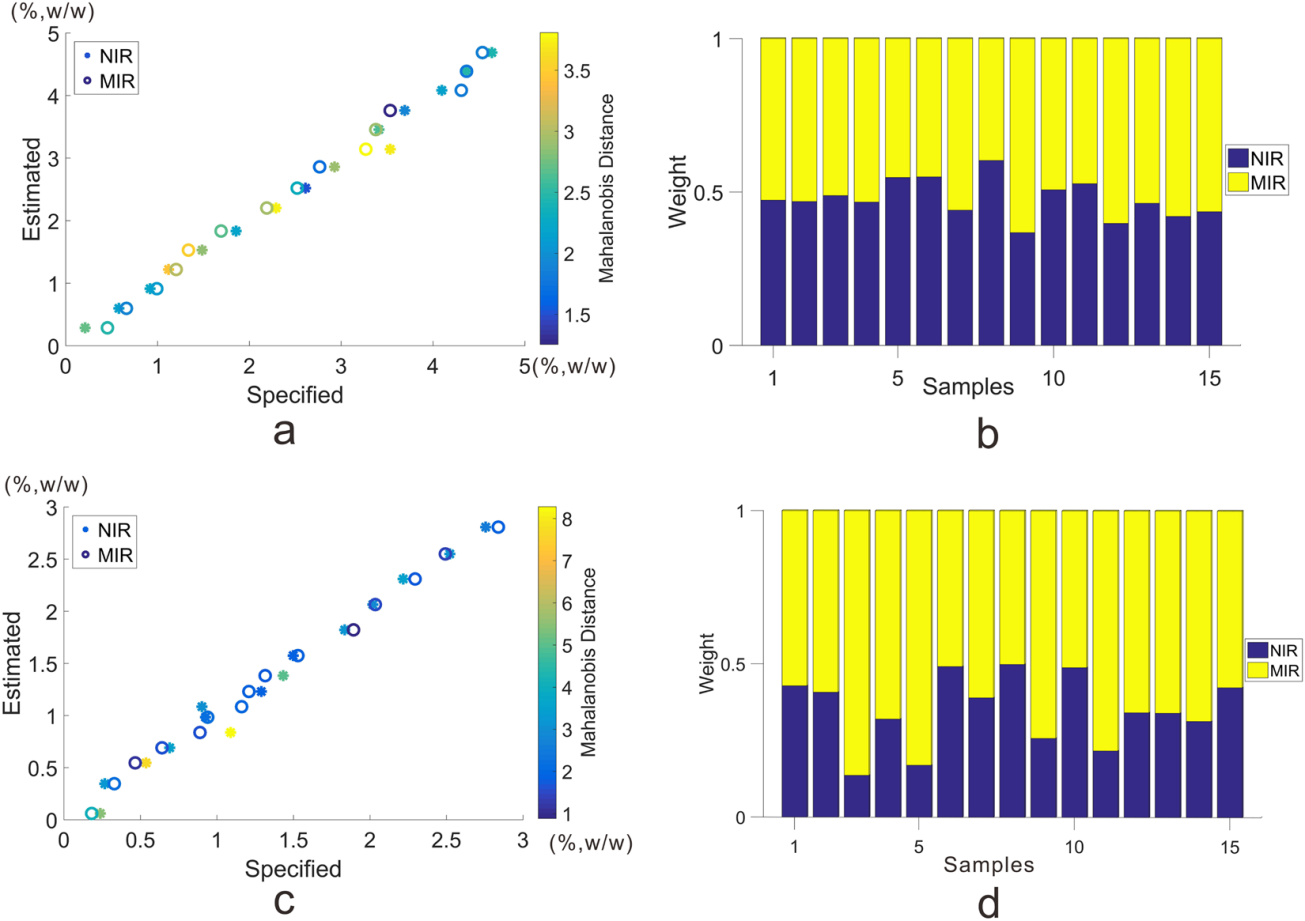
(**a**) MD values of fifteen deltamethrin samples of NIR and MIR methods; (**b**) the weights of deltamethrin samples from NIR and MIR methods for fusion; (**c**) MD values of fifteen emamectin benzoate samples of NIR and MIR methods; (**d**) the weights of emamectin benzoate samples from NIR and MIR methods for fusion.

Figure 4a evidently displayed the MD values of 15 deltamethrin samples according to NIR and MIR methods. The left y axis indicated the estimated value of validation set, and the right y axis represented the color bar of MD. As demonstrated in Fig. 4a and Table 3, the MD values were represented by different colors, the color bar was varied from dark blue to yellow indicating MD values ranged from 1.25 to 3.76 (the minimum and maximum MD values from NIR and MIR methods). Given an example, if a sample was symbolized in yellow, which indicated it owned a large MD value and meanwhile manifested that it had a farther distance from the calibration set. Thus, the corresponding method took up a smaller proportion.

Figure 4b showed the weights of deltamethrin samples, it represented the proportions occupied by NIR and MIR methods. The sum proportion of the weight equaled to 1 for one sample according to different methods, which was normalized by MD values. As shown in Fig. 4b, the sampling weights for different methods were filled with different colors, concretely speaking, the blue and yellow portions revealed the weights of NIR and MIR, respectively.

The fusion plot of estimated and specified values in validation set was displayed in Fig. 3c with the weights shown in Fig. 4b. As seen in Fig. 4b, one sample was alloted to two weights according to the performance of NIR and MIR methods. In some cases, one method might give a promising predictive performance, while the other method might not predicted well. In the circumstances, the predictability would be improved by means of the fusion strategy.

It was concluded from Table 2 that the MDW method provided an outperformed results with RMSEP of 0.1016%, bias of 0.0195% compared with the individual methods of NIR (RMSEP of 0.1164%, bias of −0.0120%) and MIR (RMSEP of 0.1294%, bias of 0.0403%). The MDW method yielded more satisfactory results than the individual technologies, which mainly declared that the MDW method was capable to generate a more accurate result than individual technologies for deltamethrin pesticide. Therefore, it was essential for deltamethrin data set to carry out MDW fusion strategy to proceed quantitative analysis. As a case to case approach to evaluate the weight according to different methods, the MDW method was feasible to employ high-level fusion for quantitative analysis.

### Emamectin benzoate formulation data set

**Figure 5:**
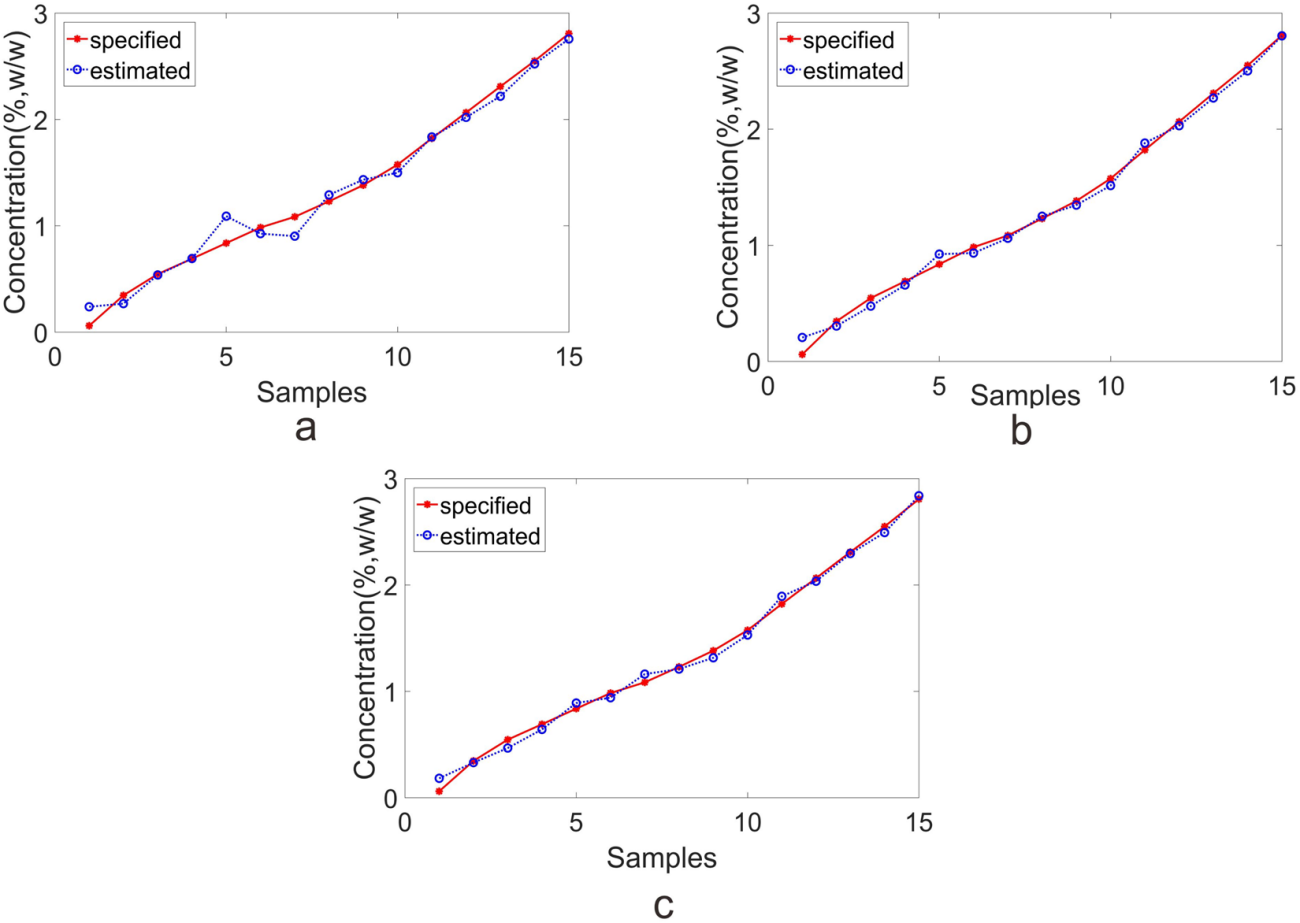
The plot of predicted and actual value in test set of emamectin benzoate samples. (**a**) Individual NIR; (**b**) individual MIR; (**c**) MDW for fusion.

MC outlier approach was carried out on the emamectin benzoate data set, and no sample was identified as the outlier. Subsequently, sixty samples were divided into calibration set (45 samples) and validation set (15 samples) according to the concentration. The emamectin benzoate data set was pre-processed by autoscaling before developing the model. After carrying out five-fold PLS algorithm, ten and four LVs were respectively chosen for individual NIR and MIR spectra method. The plot of estimated and specified values in validation set were shown in Fig. 5a,b. The predicted results for individual techniques were summarized in Table 2. As outlined in Table 2, the predictive performance of MIR (RMSEP = 0.0765%, bias = 0.0114%) gave a favorable result than that of NIR (RMSEP = 0.1047%, bias = 0.0601%).

Based on the fusion procedure, MDW method was carried out to apply high-level fusion for quantitative analysis for emamectin benzoate data set. The criteria for each method were displayed in Table 3. It was obviously obtained from Table 3 that all the samples were within the range of threshold, so the weights were assigned to the inverse ratio of the corresponding MD values. Figure 4c was the MD values of 15 samples according to different methods. As could be seen in Fig. 4c and Table 3, the MD values (0.883 to 8.283) were represented by different colors with the color bar varied from dark blue to yellow. Take a dark blue sample for instance, this sample was assigned with a comparative small MD, which in return manifesting the associated method played an important role for fusion. Figure 4d was the sampling weights, wherein the blue and yellow portions were the weights of NIR and MIR, respectively. Evidently, one sample was allocated to two weights according to MD values.

The plot of estimated and specified values in validation set were shown in Fig. 5c. The results of MDW method (RMSEP of 0.0596%, bias of 0.0205%) gained an advantage over the individual methods of NIR (RMSEP of 0.1047%, bias of 0.0601%) and MIR (RMSEP of 0.0765%, bias of 0.0114%). In summary, MDW method indeed improved the predictive ability of emamectin benzoate data set, which revealed that the MDW method was effectively to employ the individual method for fusion.

## Conclusion

In this study, the proposed MDW method was successfully applied to NIR and MIR spectroscopic analysis for rapid determination of pesticide active ingredient in deltamethrin and emamectin benzoate formulations. As a matter of fact, the MDW method performed superior results than individual NIR and MIR method mainly attributed to the fusion method took advantage of the merits of each method comprehensively. Overall, the method is promising with increased predictive ability compared with individual methods. Admittedly, the results generated by the MDW method were better than the two individual methods, indicating that the MDW method could improve the predictive ability of the model and could be successfully used for fusion.

## References

[1] Kong WJ (2016). Trace analysis of multi-class pesticide residues in chinese medicinal health wines using gas chromatography with electron capture detection. Scientific Reports.

[2] Watanabe E, Kobara Y, Baba K, Eun H (2015). Determination of seven neonicotinoid insecticides in cucumber and eggplant by water-based extraction and high-performance liquid chromatography. Analytical Letters.

[3] Li W, Sun M, Li M (2013). A survey of determination for organophosphorus pesticide residue in agricultural products. Advance Journal of Food Science and Technology.

[4] Huang Z, Li Y, Chen B, Yao S (2007). Simultaneous determination of 102 pesticide residues in Chinese teas by gas chromatography-mass spectrometry. Journal of Chromatography B.

[5] Masiá A, Blasco C, Picó Y (2014). Last trends in pesticide residue determination by liquid chromatography-mass spectrometry. Trends in Environmental Analytical Chemistry.

[6] Jamshidi B, Mohajerani E, Jamshidi J, Minaei S, Sharifi A (2015). Non-destructive detection of pesticide residues in cucumber using visible/near-infrared spectroscopy. Food Additives and Contaminants: Part A.

[7] Ozaki Y, Šašić S, Jiang J, Siesler HW (2015). Self-modeling curve resolution analysis of on‐line vibrational spectra of polymerisation and transesterification. Macromolecular Symposia.

[8] Jamshidi B, Mohajerani E, Jamshidi J (2016). Developing a Vis/NIR spectroscopic system for fast and non-destructive pesticide residue monitoring in agricultural product. Measurement.

[9] Mcsherry R (2007). Comparison of two vibrational procedures for the direct determination of mancozeb in agrochemicals. Talanta.

[10] Armenta S, Moros J, Garrigues S, Miguel DLG (2005). Automated Fourier transform near infrared determination of buprofezin in pesticide formulations. Journal of Near Infrared Spectroscopy.

[11] Tang G, Lai Y, Song X, Qiu K, Min S (2014). Rapid detection of illicit fipronil in phoxim granules by mid-infrared spectroscopy. Optik-International Journal for Light Electron Optics.

[12] Qiu K, Song X, Tang G, Wu L, Min S (2013). Determination of fipronil in acetamiprid formulation by attenuated total reflectance-mid-infrared spectroscopy combined with partial least squares regression. Analytical Letters.

[13] Ramos PM, Ruisanchez I, Andrikopoulos KS (2008). Micro-raman and X-ray fluorescence spectroscopy data fusion for the classification of ochre pigments. Talanta.

[14] Di CV, Callao MP, Ruisanchez I (2011). ^1^H NMR and UV-visible data fusion for determining Sudan dyes in culinary spices. Talanta.

[15] Ramos PM, Callao MP, Ruisanchez I (2007). Data fusion in the wavelet domain by means of fuzzy aggregation connectives. Analytica Chimica Acta.

[16] Borràs E (2015). Data fusion methodologies for food and beverage authentication and quality assessment-a review. Analytica Chimica Acta.

[17] Huang L, Zhao J, Chen Q, Zhang Y (2014). Nondestructive measurement of total volatile basic nitrogen (TVB-N) in pork meat by integrating near infrared spectroscopy, computer vision and electronic nose techniques. Food Chemistry.

[18] Roussel S, Bellon-Maurel V, Roger JM, Grenier P (2003). Fusion of aroma, FT-IR and UV sensor data based on the Bayesian inference, Application to the discrimination of white grape varieties. Chemometrics and Intelligent Laboratory Systems.

[19] Kuncheva LI, Whitaker CJ, Shipp CA, Duin RPW (2003). Limits on the majority vote accuracy in classifier fusion. Pattern Analysis and Applications.

[20] Zhang R, Ji Q (2006). Active and dynamic information fusion for multisensor systems with dynamic bayesian networks. IEEE Transactions on Systems, Man, and Cybernetics, Part B (Cybernetics).

[21] Jerome JB (2000). Dempster-Shafer theory and Bayesian reasoning in multisensor data fusion. Proceedings of SPIE-The International Society for Optical Engineering.

[22] Arandasanchez JI, Baltazar A, Gonzálezaguilar G (2009). Implementation of a bayesian classifier using repeated measurements for discrimination of tomato fruit ripening stages. Biosystems Engineering.

[23] Li C, Heinemann P, Sherry R (2007). Neural network and bayesian network fusion models to fuse electronic nose and surface acoustic wave sensor data for apple defect detection. Sensor and Actuators B: Chemical.

[24] Roussel S, Bellon-Maurel V, Roger JM, Grenier P (2003). Fusion of aroma, FT-IR and UV sensor data based on the bayesian inference application to the discrimination of white grape varieties. Chemometrics and Intelligent Laboratory Systems.

[25] Baltazar A, Aranda JI, González-Aguilar G (2008). Bayesian classification of ripening stages of tomato fruit using acoustic impact and colorimeter sensor data. Computers and Electronics in Agriculture.

[26] Zou X, Zhao J (2005). Apple quality assessment by fusion three sensors. Journal of IEEE Sensor.

[27] Li R, Wang P, Hu W (2000). A novel method for wine analysis based on sensor fusion technique. Sensor and Actuators B: Chemical.

[28] Mahalanobis PC (1936). On the generalized distance in statistics. Proceedings of the National Institute of Sciences of India.

[29] Mark HL, Tunnell D (1985). Qualitative near-infrared reflectance analysis using Mahalanobis Distances. Analytical Chemistry.

[30] Whitfield RG, Gerger ME, Sharp RL (1987). Near-infrared spectrum qualification via Mahalanobis distance determination. Applied Spectroscopy.

